# Navigator gated flow sensitive gradient echo sequence for quantification of aortic valve regurgitation

**DOI:** 10.1186/1532-429X-16-S1-P266

**Published:** 2014-01-16

**Authors:** Jonathan Nadjiri, Caroline Sonne, Eva Hendrich, Albrecht Will, Cornelia Pankalla, Stefan Martinoff, Martin Hadamitzky

**Affiliations:** 1 Department of Radiology and Nuclear Medicine, Deutsches Herzzentrum München, Munich, Bavaria, Germany; 2 Department of Cardiology, Deutsches Herzzentrum München, Munich, Germany

## Background

Exact quantification of aortic valve regurgitation is a challenge. Patients are typically asymptomatic and echocardiography is unreliable in certain conditions, particularly in eccentric regurgitation jets. Cardiac magnetic resonance (CMR) is a valuable alternative in this situation, but the use of conventional sequences without respiration correction is limited by motion artifacts and long acquisition times caused by necessary signal averaging. Novel navigator gated flow sensitive gradient echo sequence avoid motion artifacts caused by respiration and in addition allow for a higher temporal resolution eventually leading to more reliable results.

## Methods

On 31 patients with varying degrees of aortic regurgitation we performed both standard (StdSeq) and navigator gated (NavSeq) flow sensitive gradient echo sequences. Primary parameter was the regurgitation fraction defined as the quotient of anterograde and retrograde flow over a cross section of the ascendent aorta just above the maximal excursion of the aortic leaflets. Since a gold standard for quantification of aortic regurgitation is missing, we correlated these values with the enddiastolic volume of the left ventricle (LVEDV) assessed by a contiguous stack short axis cine-SSFP sequences. This parameter is directly influenced by the regurgitant volume. For comparison we recorded the aortic regurgitation in echocardiography using 5 degrees (mild - mild to moderate - moderate - moderate to severe -severe ) assessed by an expert echocardiographer blinded to the CMR results.

## Results

**Figure 1:**
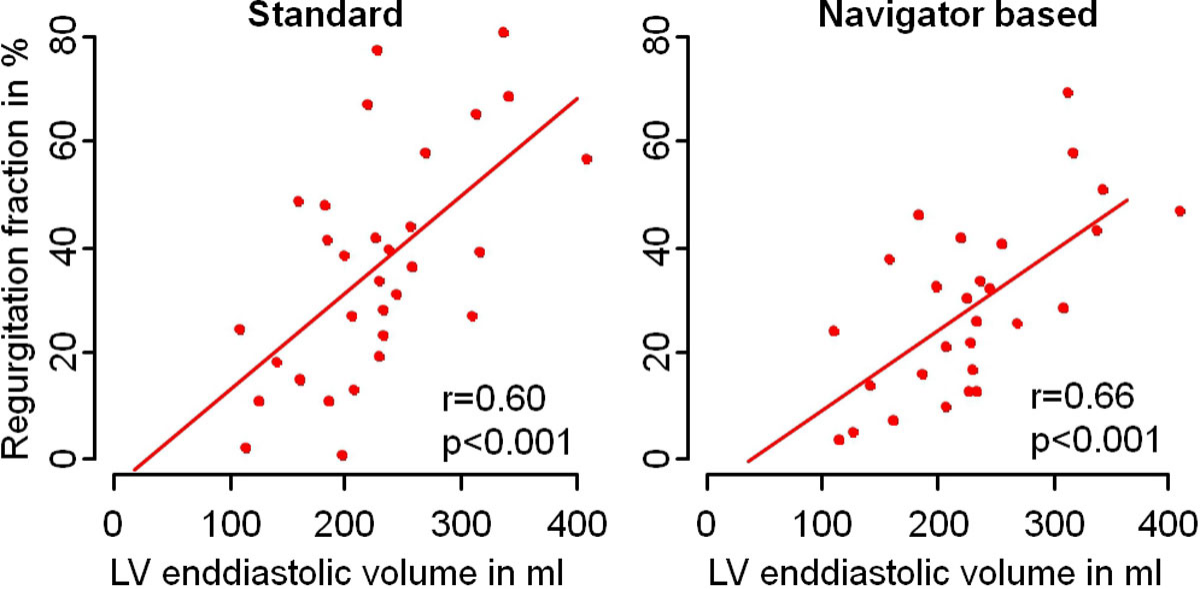


Regurgitation fraction ranged from 0% to 80% and from 3% to 70% in StdSeq and NavSeq, respectively. Values of LVEDV ranged between 109 and 409 ml. NavSeq correlated best with LVEDV with a slope of 0.15 percentage points per ml increase and a correlation coefficient r of 0.66 (p < 0.001). For StdSeq the values of r and slope were 0.60 (p < 0.001) and 0.18, respectively (Figure 1 below). Correlation with echocardiographic assessment of aortic regurgitation was weak with a p value of 0.03 and 0.01 for NavSeq and StdSeq, respectively, as was the correlation between the echo quantification and LVEDV (p = 0.012).

## Conclusions

Navigator gated flow quantification had a better correlation with LVEDV than the conventional non-gated sequence. The correlation to LVEDV of both CMR sequences is better than that of echocardiography. Novel navigator gated flow sensitive gradient echo sequence further improves accuracy of quantification of aortic regurgitation, this may be a valuable alternative modality for patients with inconclusive echocardiographic findings.

## Funding

None.

